# High prevalence of pre-treatment HIV drug resistance in Papua New Guinea: findings from the first nationally representative pre-treatment HIV drug resistance study

**DOI:** 10.1186/s12879-022-07264-y

**Published:** 2022-03-19

**Authors:** Janet Gare, Ben Toto, Percy Pokeya, Linh-Vi Le, Nick Dala, Namarola Lote, Bangan John, Abel Yamba, Kevin Soli, Joshua DeVos, Heather Paulin, Nick Wagar, Du-Ping Zheng, Takeshi Nishijima, Peniel Boas, Angela Kelly-Hanku, Anup Gurung

**Affiliations:** 1 Sexual and Reproductive Health Unit, PNG Institute of Medical Research, Corner of Leigh Vial & Homate St, Goroka, Eastern Highlands Province 441 Papua New Guinea; 2 United States Centres for Disease Control and Prevention Country Office of Papua New Guinea, Port Moresby, National Capital District Papua New Guinea; 3 HIV, Hepatitis and STI Unit, Division of Communicable Disease, World Health Organization Regional Office of the Western Pacific, Manila, Philippines; 4 Director’s Office, National AIDS Council Secretariat, Port Moresby, National Capital District Papua New Guinea; 5 World Health Organization Country Office of Papua New Guinea, Port Moresby, National Capital District Papua New Guinea; 6 Division of Global HIV and TB, International Lab Branch, United States Centers for Disease Control and Prevention, Atlanta, GA USA; 7 Division of Global HIV and TB, HIV Care and Treatment Branch, United States Centers for Disease Control and Prevention, Atlanta, GA USA; 8 National Center for Global Health and Medicine, Tokyo, Japan; 9 National HIV Program Division, National Department of Health, Port Moresby, National Capital District Papua New Guinea; 10 Kirby Institute for Infection and Immunity in Society, University of New South Wales, Sydney, NSW Australia

**Keywords:** Human immunodeficiency virus, Pre-treatment drug resistance, Antiretroviral therapy, Papua New Guinea

## Abstract

**Background:**

Determining the prevalence of pre-treatment HIV drug resistance (PDR) is important to assess the effectiveness of first-line therapies. To determine PDR prevalence in Papua New Guinea (PNG), we conducted a nationally representative survey.

**Methods:**

We used a two-stage cluster sampling method to recruit HIV treatment initiators with and without prior exposure to antiretroviral therapies (ART) in selected clinics. Dried blood spots were collected and tested for PDR.

**Results:**

A total of 315 sequences were available for analysis. The overall PDR prevalence rate was 18.4% (95% CI 13.8–24.3%). The prevalence of PDR to non-nucleoside analog reverse-transcriptase inhibitors (NNRTIs) was 17.8% (95% CI 13.6–23.0%) and of PDR to nucleoside reverse transcriptase inhibitors (NRTIs) was 6.3% (95% CI 1.6–17.1%). The PDR prevalence rate among people reinitiating ART was 42.4% (95% CI 29.1–56.4%).

**Conclusions:**

PNG has a high PDR prevalence rate, especially to NNRTI-based first-line therapies. Our findings suggest that removing NNRTIs as part of first-line treatment is warranted and will lead to improving viral suppression rates in PNG.

## Background

Global antiretroviral therapy (ART) scale-up in the last 15 years has averted millions of AIDS-related deaths; however, there is evidence of increasing prevalence of pre-treatment drug resistance (PDR) to a non-nucleoside reverse transcriptase inhibitor (NNRTI)-based treatment regimen [1]. PDR can possibly increase HIV incidence and AIDS-related mortality rates [2, 3] and has direct multifaceted implications on national and global HIV care and treatment programs.

With a population of more than 8 million, PNG has a complex HIV epidemic, concentrated in key populations, namely female sex workers and men who have sex with men and transgender women and in particular geographical areas such as the highlands and southern regions [4, 5]. At around the time of the study (Dec 2018) approximately 45,000 people living with HIV (PLHIV); 29,420 were on ART of the end of 2018 [6], 90% received a combination of tenofovir (TDF) or zidovudine plus lamivudine and efavirenz or nevirapine. While considerable progress has been made to initiate PLHIV on ART, around only 70% are retained in care in a given year [7]. HIV treatment outcome monitoring in PNG relies on clinical presentation and CD4+ T-cell counts. While attempts are being made to scale up HIV viral load monitoring, such testing is still in its infancy and not yet at scale or provided at point-of-care. Routine HIV drug resistance (HIVDR) testing is not available anywhere in the country. The risk of HIVDR is further compounded by ongoing shortages of ART in the country.

The data on HIVDR in PNG is limited. Since the availability of ART in PNG in 2004, two HIVDR studies have been conducted. The first study, conducted in 2009 in two major ART clinics in the highlands of PNG, reported 2.1% (N = 96) prevalence of resistance to NNRTIs among ART-naïve patients [8]. In 2010, a transmitted drug resistance study among adults aged 15–30 years recently infected with HIV and ART- naïve revealed concerning levels of resistance to NNRTIs; 16.1% in the capital Port Moresby and 8.2% in Mt. Hagen, Western Highlands Province [9]. To guide policy and treatment guidelines, we conducted a nationally representative survey to estimate the national prevalence of PDR in PNG.

## Methods

### Study design

A cross-sectional survey using a two-stage cluster sampling design based on the 2014 World Health Organization (WHO)  concept note on surveillance of HIVDR in adults initiating ART was undertaken from July 2017 to April 2018[10]. We first selected ART clinics using probability proportional to size sampling and then we recruited patients who met the eligibility criteria at each selected clinic as they register into HIV care. The survey included 14 ART clinics (one clinic was sampled twice) across eight provinces. The target estimated sample size was calculated at 345 (23 per clinic except the clinic that was sampled twice had 46) following the sampling method suggested in the 2014 WHO PDR concept note [10].

### Patient selection

Eligibility criteria included being HIV positive, being aged ≥ 18 years, providing written informed consent, and either initiating ART with prior ART exposure (through prophylactic treatment including mothers on Option B+) or without prior ART exposure) or re-initiating ART (first or second line therapy) after a treatment interruption of ≥ 90 days.

### Specimen collection, handling, and processing

Specimen collection, handling, and processing procedures followed the WHO HIVDR guidelines using dried blood spots (DBS) for HIVDR testing [11]. Consenting participants provided 500 µL of venous whole blood, which was spotted on Whatman 903 filter paper cards. DBS were dried at room temperature and sent by courier within three days of collection to central laboratories in Goroka and Port Moresby and stored at − 80 °C. DBS were shipped to a WHO-accredited laboratory at US CDC in Atlanta, GA, for HIVDR testing.

### HIVDR genotyping

The protease and reverse transcriptase regions of the HIV-1 *pol* gene were genotyped [12]. Sequencing was performed using the Thermo Fisher (Waltham, MA, USA) HIV-1 Genotyping Kit Amplification and Cycle Sequencing Module Kit and analyzed on an ABI 3730 DNA Analyzer (Applied Biosystems; Waltham, MA, USA). The ReCALL software was used to edit the raw sequences and generate consensus sequences [13]. Sequence quality assurance was performed using genetic pairwise distances (2% cut-off) and neighbour joining tree analyses, performed in MEGA to rule out possible sample contamination [14]. Sequences were classified as having low-level, intermediate, or high-level resistance according to the Stanford HIV database algorithm (version 8.5) and were aggregated as “HIV drug resistance”.

### HIV PDR prevalence analysis

Data were analyzed using Stata version 14, StataCorp, Texas, USA. PDR prevalence rates were calculated at the national level and stratified by prior ART exposure status with respective confidence intervals. Some of the analyses were adjusted for survey weights and clustering.

### Ethics

The study received ethics clearance from the Ethics Review Committee of the Western Pacific Regional Office of World Health Organization (2015.52.PNG.2.HSI), PNG Medical Research Advisory Committee (16.39) and PNG Institute of Medical Research Institutional Review Board (1605). This project was also reviewed in accordance with United States Centres for Disease Control and Prevention (US CDC) human research protection procedures. Informed consent was obtained from all the study participants.

## Results

### Demographic and clinical characteristic of patients

**Fig. 1:**
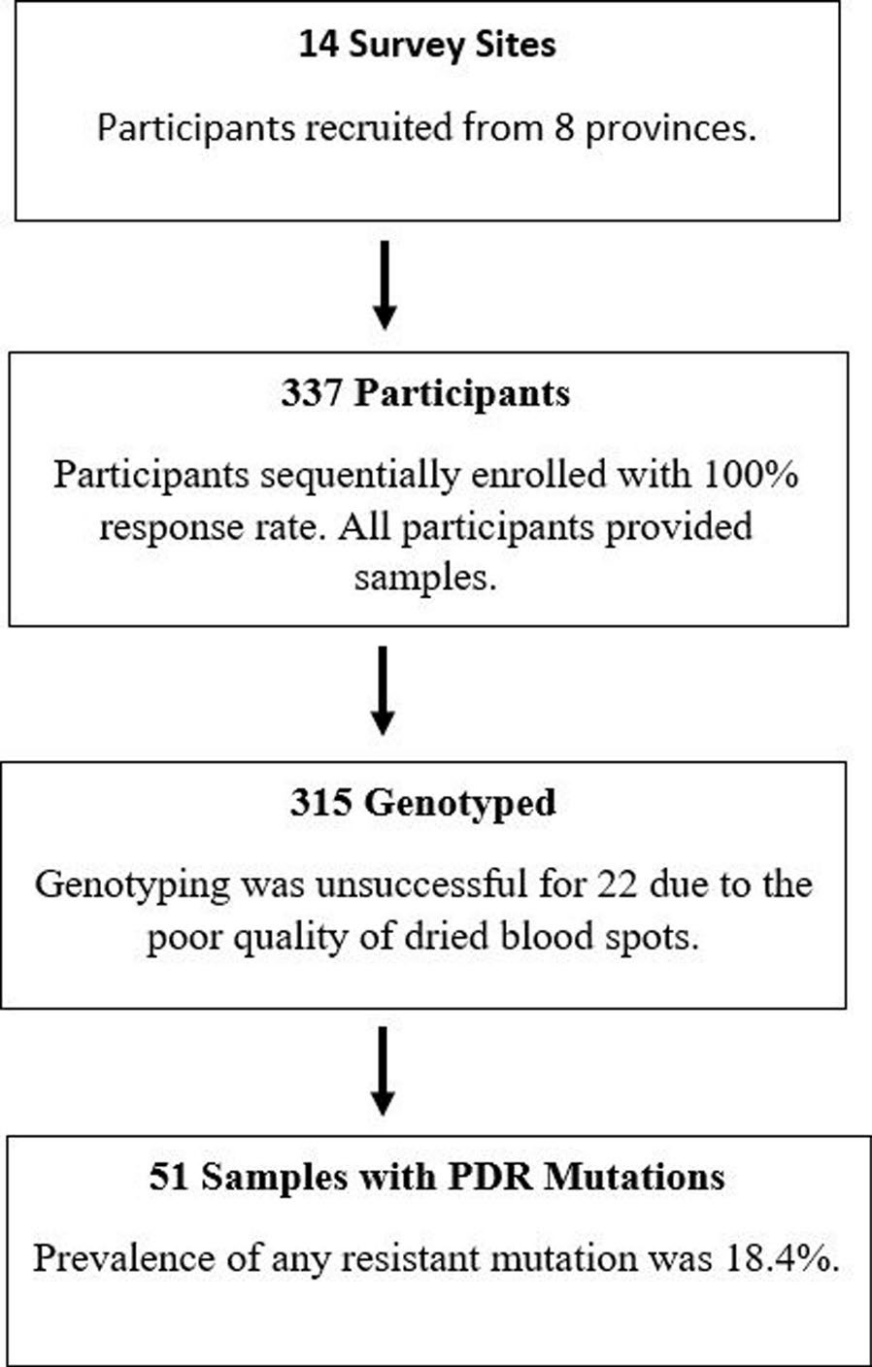
The flow chart illustrates the study participant recruitment process and the genotyping results

The summary of participant recruitment and drug resistance analysis is depicted in Fig. 1. Of the 337 of participants enrolled; 268 (79.5%) were ART naïve and 69 (20.5%) had previous exposure to ART. All of those who had previous exposure to ART had interrupted treatment with none through PMTCT or pre-exposure prophylaxis. Most (207; 61.4%) of participants were female. The mean age was 31 years (range 18–67 years) (Table 1). Of the 236 (70%) participants with CD4 T-cell counts, the mean was 237 cells/mm^3^ (range 10–121 cells/mm^3^), and 61.9% had CD4-T-cell counts ≤ 250 cells/mm^3^ (Table 1). Of the 323 participants who had WHO Disease Staging assessed, 172 (53.3%) had stage III disease. Of the 312 participants who initiated first-line ART, 84% (n = 267) received a TDF-based regimen (Table 1). There were 25 participants that had no written records of first-line ART initiated either due to shortage of ART or they were treated for other infections and ART initiation was delayed at the time of survey.

**Table 1:** Clinical characteristics of patients with HIV who participated in a national survey to determine prevalence of HIV drug resistance in Papua New Guinea (July 2017–April 2018)

Characteristics	n	%	(95% CI)
Sex	337		100
Male	128	37.0	(33.0–41.3)
Female	207	62.5	(57.9–66.8)
Unknown	2	0.5	(0.1–2.3)
Mean age, years	31.0		(29.6–32.4)
ART status	337	100	
Naïve	268	79.1	(69.7–86.2)
Previous exposure to ART	69	20.9	(13.8–30.0)
WHO clinical staging^a^	323	100	
I	64	19.8	–
II	74	22.9	–
III	172	51.3	–
IV	13	4.0	–
CD4 cell counts^a^	236	100	
≤ 250 cells/mm^3^	146	61.9	–
> 250 cells/mm^3^	90	38.1	–
NNRTI-based first-line ART	312	100	
TDF-based	267	84.0	(63.1–94.2)
AZT-based	43	15.4	(5.7–35.4)
d4T-based	2	0.5	(0.1–3.5)

*CI* confidence interval, *ART* antiretroviral therapy, *WHO* World Health Organization, *NNRTI* non-nucleoside reverse transcriptase inhibitors, *TDF* tenofovir, *AZT* zidovudine, *d4T* stavudine

^a^Presented as un-weighted proportions rest of parameters with confidence intervals are study weighted proportions

### Patient drug resistance profile

**Fig. 2:**
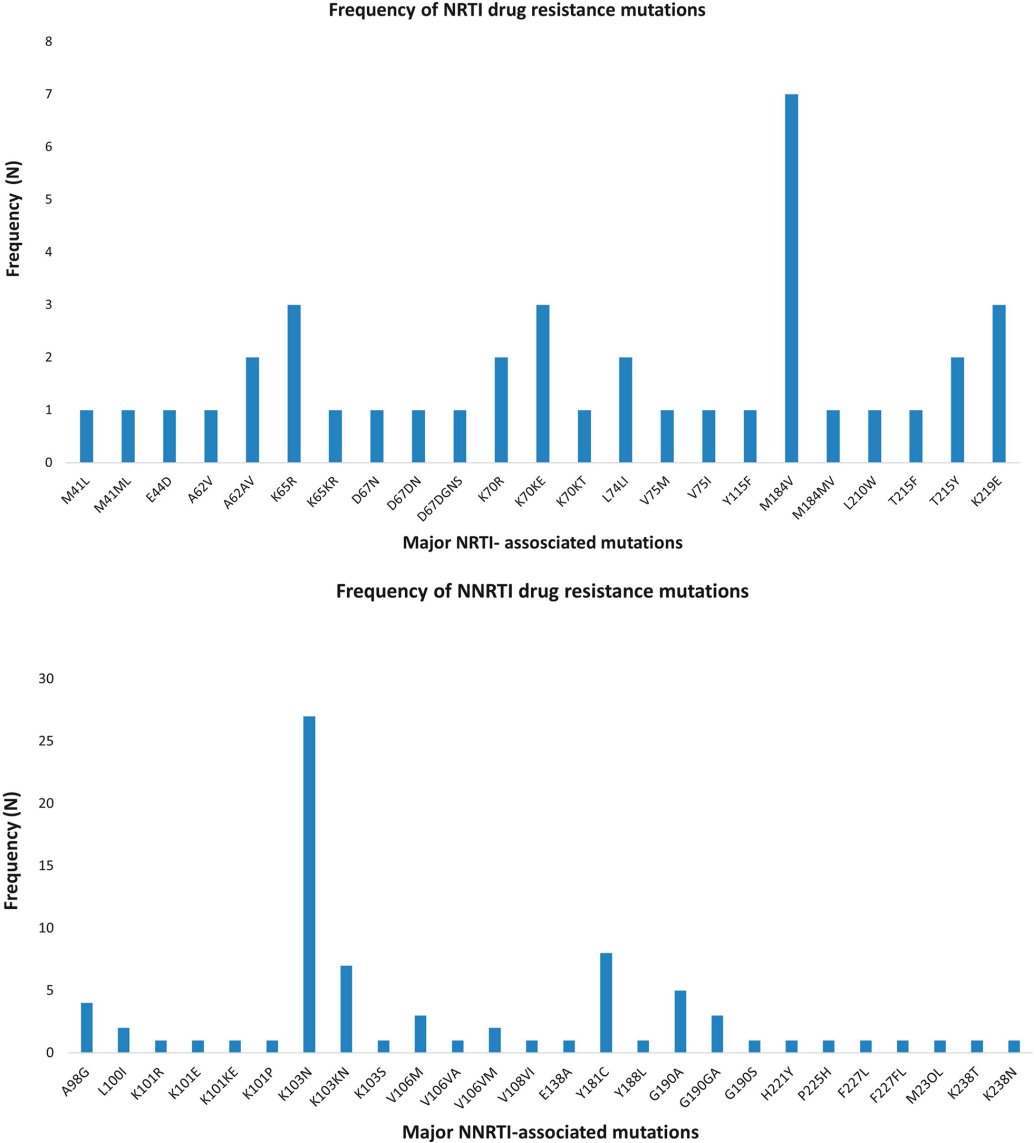
**a** Individual NRTI PDR mutations that were detected in 13 of the participants whilst. **b** Individual NNRT PDR mutations detected in 49 of the participants

Of the total 337 DBS, 315 were successfully amplified with sequences available for analysis whilst 22 failed to amplify. PDR surveillance drug resistance mutations were detected in 51/315 of patients yielding an overall prevalence of any resistance mutations at 18.4% (95% CI 13.8–24.3%) (Table 2). NNRTI-related PDR mutations occurred in 17.8% (95% CI 13.6–23.0) of sequences whilst NRTI-related occurred in 5.6% (95% CI 1.6–17.1%). Eleven patients had SDRMs to both NNRTIs and NRTIs. There were no protease inhibitor-resistant mutations. The most frequent occurring NNRTI mutation was *K103N/K103KN* followed by *Y181C*, *M184V* was the most prevalent NRTI mutation (See Fig. 2a and b). All sequences were subtype C except one was subtype A and another B. Of note, on CDC laboratory routine quality checks 59/315 samples showed > 98% genetic similarity, which indicates high homology.

**Table 2:** HIV Pre-treatment drug resistance prevalence among people with HIV initiating and re-initiating ART in Papua New Guinea (July 2017–April 2018), by first-line treatment drug classes

All patients initiating or re-initiating ART		
	N = 315	% (95% CI)^a^
Any	51	18.4 (13.8–24.3)
NNRTI^2^	49	17.8 (13.6–23.0)
NRTI	13	5.6 (1.6–17.1)
PI^3^	0	0.0 (0.0–1.2)
NNRTI + NRTI	11	4.9 (1.5–14.5)

Patients initiating who are ART naïve		
	n = 254	% (95% CI)^a^
Any	30	12.3 (7.8–18.9)
NNRTI	28	11.6 (7.0–18.5)
NRTI	6	2.7 (1.0–7.1)
PI	0	0.0 (0.0–1.2)
NNRTI + NRTI	4	1.8 (0.6–5.9)

Patients re-initiating ART (ART-exposed)		
	n = 61	% (95% CI)^a^
Any	21	42.4 (29.1–56.9)
NNRTI	21	42.4 (29.1–56.9)
NRTI	7	16.9 (4.1–49.1)
PI	0	0.0 (0.0–1.2)
NNRTI + NRTI	7	16.9 (4.1–49.1)

*CI* confidence interval, *ART* antiretroviral therapy, *NNRTI* non-nucleoside reverse transcriptase inhibitors, *NRTI* nucleoside reverse transcriptase inhibitor; *PI* protease inhibitor

^a^Study design-weighted proportion and 95% confidence interval; ^2^NNRTI-based first-line regimens include efavirenz or nevirapine; ^3^PI-based first-line regimens include atazanavir, darunavir, or lopinavir/ritonavir

## Discussion

Our study is the first nationally representative survey of PDR, and our findings have important implications for the national HIV response in PNG. The overall prevalence rate of the frequency of PDR mutations is high (18.4%) as is the PDR mutations to NNRTIs (17.8%); placing PNG among the top five countries with PDR rates exceeding > 10% [15]. As per the *Global Action Plan on HIV Drug Resistance 2017–2021*, countries reporting NNRTI-resistance prevalence of > 10% are recommended to change from an NNRTI to a non-NNRTI-based first-line ART regimen [16]. WHO now highly recommends dolutegravir (DTG), a potent integrase inhibitor with a high genetic barrier to HIVDR as the preferred first-line drug [17]. Additionally, DTG has minimal side effects, is affordable, and is increasingly available in many low- and middle-income countries: [18, 19] A DTG-containing regimen remains the most affordable treatment option for patients in PNG. Subsequent to the findings of this study, and the treatment advocacy that it resulted in, the Government of PNG procured new first line treatment and has, with its development partners, commenced ongoing transition to DTG.

With overall NNRTI-resistance prevalence rates of 17.8%, and 42.4% (Table 2) among the treatment-experienced, our findings suggest that NNRTI-containing regimens should be removed from use as first-line treatment in PNG. Changing the national treatment guidelines could halt HIVDR emergence. In addition to guideline changes and rolling out of DTG-based regimens, improvements in PNG’s health system, including improving availability of HIV drugs both at the national and facility level, efficient drug distribution systems to avoid stock outs, reducing attrition rates after 12 months on ART, and improving treatment adherence could help improve patient outcomes.

With the absence of routine HIVDR testing in PNG, HIV viral load monitoring could be useful in identifying early signs of adherence issues and/or treatment failure. Increased efforts are needed for an expansion of a quality HIV viral load testing program throughout the country, including providing such testing at point-of-care. In the absence of routine HIVDR testing if HIV viral load testing was widely available across the entire country (and people got their results in a timely manner) this could assist in understanding the emergence of further PDR. Our study showed that over half of the participants are presenting to clinics in the late stages (symptomatic) of the course of HIV infection and getting tested for HIV. This poses a challenge to the efforts to fast track the first 95 UNAIDS target where 95% of people who are living with HIV to know their HIV status. Although, HIV testing is available in the most parts of PNG, the uptake of HIV testing is poor outside of the clinical settings.

Our findings provide insight into PDR rates in PNG’s adult population, but no survey has been conducted for children, infants, or pregnant women enrolled and treated for HIV within prevention of parent-to-child transmission programs. Future HIVDR research must be expanded to include children and pregnant women. With a concentrated HIV epidemic among key populations in PNG [4, 5], it will be critical to determine HIVDR within these populations as HIVDR will adversely affect efforts to address the last target of the global aim to End AIDS where 95 of people have suppressed HIV viral load. As it is, we were not able to determine people’s membership of key populations in our study. Finally, our findings reported high-homology samples; a further analysis of high-homology samples is ongoing to better understand possible transmission networks.

## Conclusion

In conclusion, increased efforts, including the introduction of DTG as the preferred first-line drug and improving supply chain could improve treatment adherence, retention, and reduction of transmission of PDR. Moreover, expanding and incorporating DTG transition and viral load testing into routine HIV care is likely to result in reduced PDR rates and improve patient outcomes for people living with HIV in PNG.

## Data Availability

The datasets used and/or analysed during the current study are available in the National Centre for Biotechnology Information GenBank. GenBank accession numbers OM176712-OM177027.

## Abbreviations

ART: Antiretroviral therapy
AZT: Zidovudine
DBS: Dried blood spots
DTG: Dolutegravir
d4T: Stavudine
HIV: Human immunodeficiency virus
HIVDR: Human immunodeficiency virus drug resistance
NNRTI: Non-nucleoside reverse transcriptase inhibitors
NRTI: Nucleoside reverse transcriptase inhibitor
PDR: Pre-treatment drug resistance
PI: Protease inhibitor
TDF: Tenofovir
